# Supplementary material for: A Humanized Yeast Phenomic Model of Deoxycytidine Kinase to Predict Genetic Buffering of Nucleoside Analog Cytotoxicity
Source: Genes (Basel). 2019 Sep 30;10(10):770. doi: 10.3390/genes10100770 (PMC6826991; doi:10.3390/genes10100770)
Supplement: Supplementary file 1 [file genes-10-00770-s001.zip › Additional_Files/Additional_File7_GOTermHeatmaps.zip!/Additional_File7_GOTermHeatmaps/maturation_of_5.8S_rRNA.pdf]

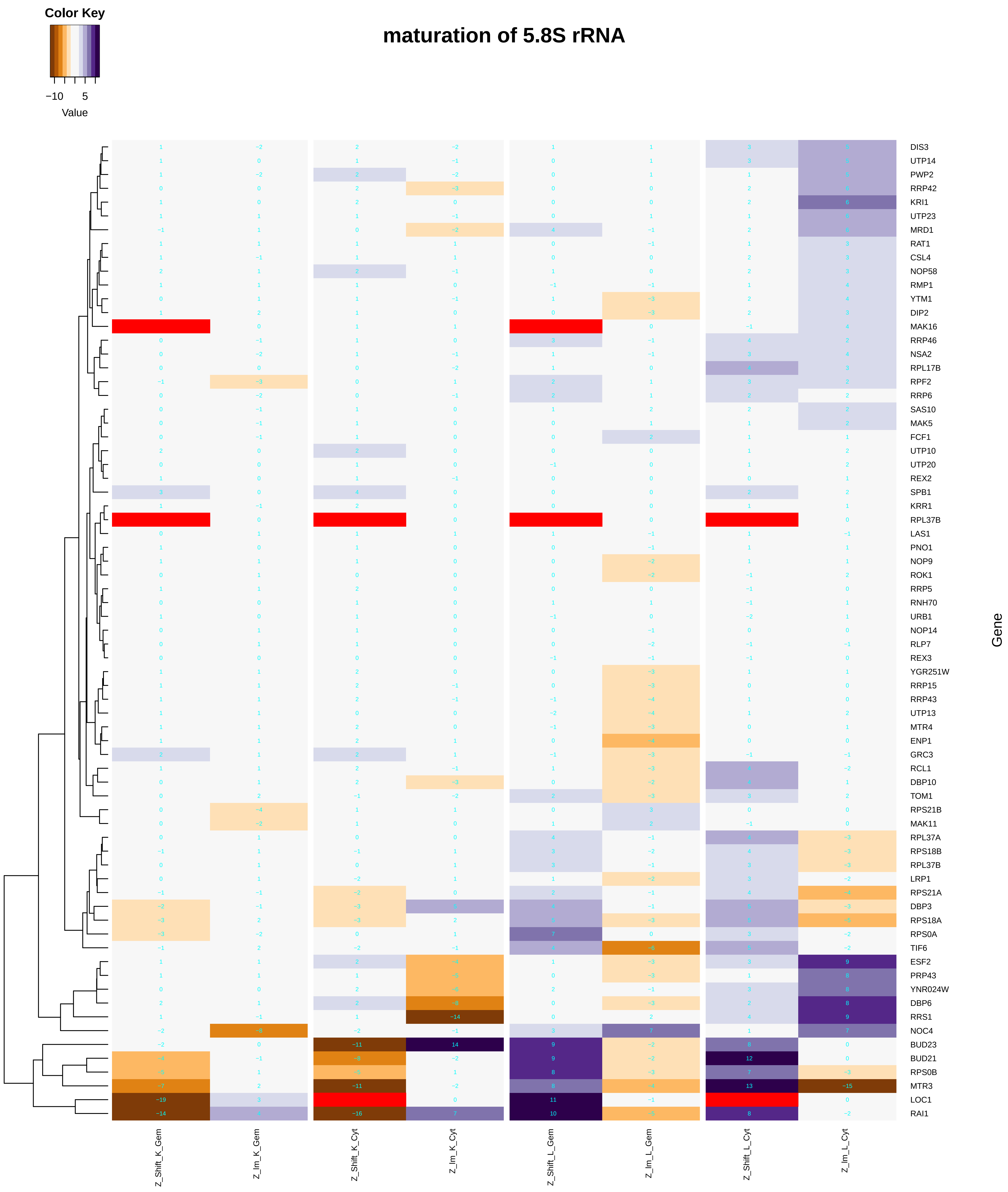

Color Key
−10
5
Value
maturation of 5.8S rRNA
DIS3
UTP14
PWP2
RRP42
KRI1
UTP23
MRD1
RAT1
CSL4
NOP58
RMP1
YTM1
DIP2
MAK16
RRP46
NSA2
RPL17B
RPF2
RRP6
SAS10
MAK5
FCF1
UTP10
UTP20
REX2
SPB1
KRR1
RPL37B
LAS1
PNO1
NOP9
ROK1
RRP5
RNH70
URB1
NOP14
RLP7
REX3
YGR251W
RRP15
RRP43
UTP13
MTR4
ENP1
GRC3
RCL1
DBP10
TOM1
RPS21B
MAK11
RPL37A
RPS18B
RPL37B
LRP1
RPS21A
DBP3
RPS18A
RPS0A
TIF6
ESF2
PRP43
YNR024W
DBP6
RRS1
NOC4
BUD23
BUD21
RPS0B
MTR3
LOC1
RAI1
Gene
Z_Shift_K_Gem
Z_Im_K_Gem
Z_Shift_K_Cyt
Z_Im_K_Cyt
Z_Shift_L_Gem
Z_Im_L_Gem
Z_Shift_L_Cyt
Z_Im_L_Cyt

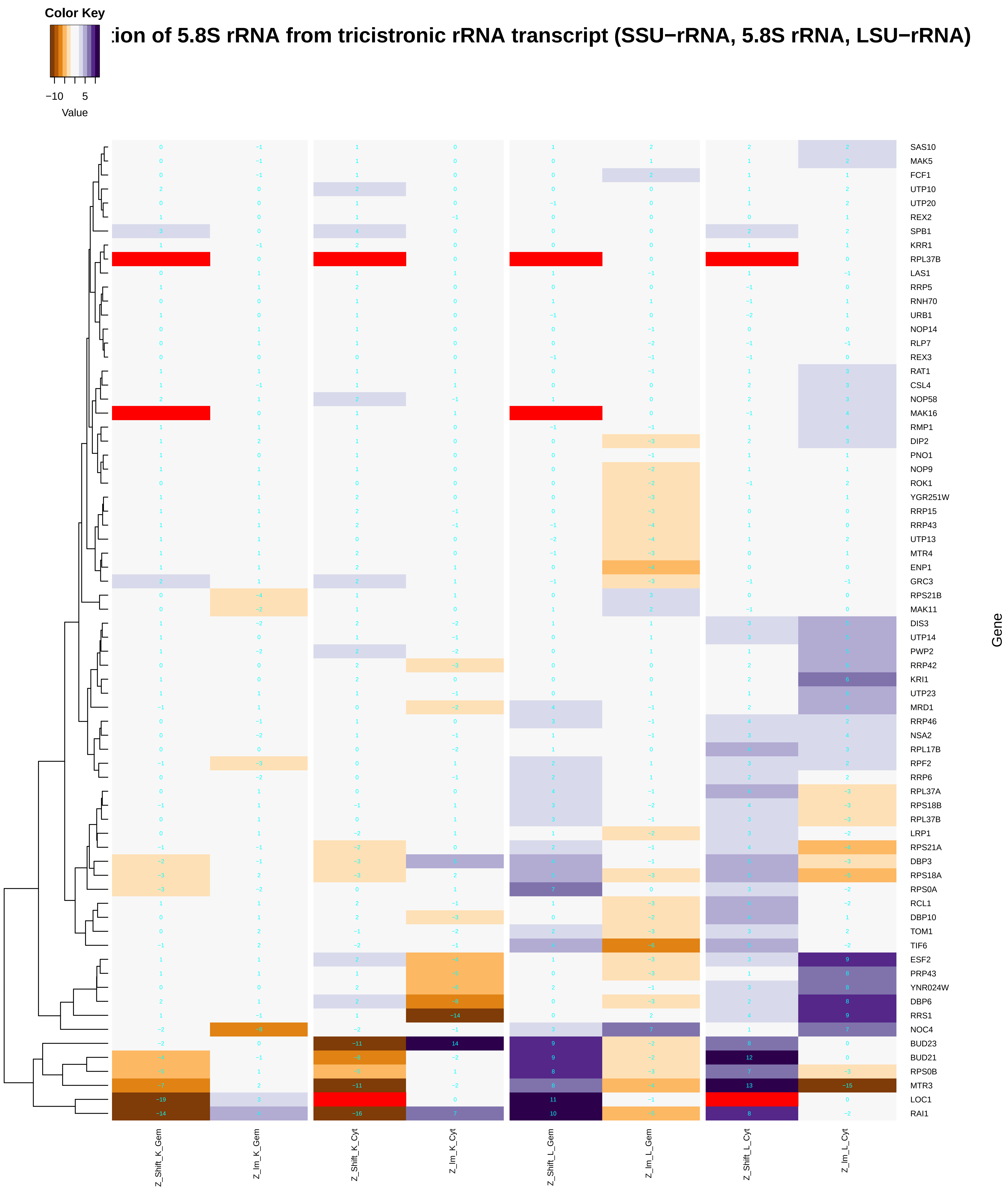

Color Key
−10 5
Value
:ion of 5.8S rRNA from tricistronic rRNA transcript (SSU−rRNA, 5.8S rRNA, LSU−rRNA)
SAS10
MAK5
FCF1
UTP10
UTP20
REX2
SPB1
KRR1
RPL37B
LAS1
RRP5
RNH70
URB1
NOP14
RLP7
REX3
RAT1
CSL4
NOP58
MAK16
RMP1
DIP2
PNO1
NOP9
ROK1
YGR251W
RRP15
RRP43
UTP13
MTR4
ENP1
GRC3
RPS21B
MAK11
DIS3
UTP14
PWP2
RRP42
KRI1
UTP23
MRD1
RRP46
NSA2
RPL17B
RPF2
RRP6
RPL37A
RPS18B
RPL37B
LRP1
RPS21A
DBP3
RPS18A
RPS0A
RCL1
DBP10
TOM1
TIF6
ESF2
PRP43
YNR024W
DBP6
RRS1
NOC4
BUD23
BUD21
RPS0B
MTR3
LOC1
RAI1
Gene
Z_Shift_K_Gem
Z_Im_K_Gem
Z_Shift_K_Cyt
Z_Im_K_Cyt
Z_Shift_L_Gem
Z_Im_L_Gem
Z_Shift_L_Cyt
Z_Im_L_Cyt
